# Tracheal Complications Following Prolonged Invasive Ventilation in Tracheostomized Pediatric Patients with Complex Chronic Conditions

**DOI:** 10.3390/children12060769

**Published:** 2025-06-13

**Authors:** Dejan Vlajnic, Deborah Wiesmann, Jens Ortmann, Mandira Reuther, Boris Zernikow

**Affiliations:** 1 Pediatric Palliative Care Centre, Children’s and Adolescents’ Hospital Datteln, 45711 Datteln, Germany; d.wiesmann@kinderklinik-datteln.de (D.W.); j.ortmann@kinderklinik-datten.de (J.O.); m.reuther@kinderklinik-datteln.de (M.R.); b.zernikow@kinderklinik-datteln.de (B.Z.); 2 Department of Children’s Pain Therapy and Pediatric Palliative Care, Faculty of Health, School of Medicine, Witten/Herdecke University, 58455 Witten, Germany

**Keywords:** tracheostomy, long-term ventilation, home ventilation, tracheoscopy, bronchoscopy, children, pediatric, palliative care, complex chronic conditions

## Abstract

This case series presents four pediatric patients who developed tracheal complications after prolonged invasive mechanical ventilation. The first case involved an 11-year-old girl with severe hypoxic encephalopathy who developed extensive ulcerative granulation tissue obstructing 60% of the tracheal lumen. The second case was that of a 6-year-old boy with ACTA1-related nemaline myopathy who experienced recurrent tracheal obstruction due to granulomatous tissue formation. The third case involved a 9-year-old boy with hydroxyglutaric aciduria and a large plug obstructing his trachea. The last case involved a 19-year-old female with lissencephaly who developed a tracheoesophageal fistula. These cases highlight the importance of regular surveillance and early intervention in managing tracheal complications in pediatric patients with complex chronic conditions requiring long-term mechanical ventilation. The authors emphasize the need for specialized care and routine endoscopic examinations in order to prevent and address potentially life-threatening complications in this vulnerable patient population.

## 1. Introduction

Tracheal complications arising from tracheotomies and prolonged invasive mechanical ventilation are often observed in pediatric patients [1,2,3,4], and they can occasionally have severe or even life-threatening consequences [5].

The reasons why a tracheostomy might be performed on a pediatric patient include providing prolonged airway and ventilatory support [6,7]. A tracheostomy is a common surgical procedure used to treat critically ill children [6]. The indications for performing a tracheostomy on a child are changing, with the most common indication now being prolonged ventilation [7]. Compared to adults [8], children face greater technical difficulties during a tracheostomy and during post-tracheostomy care, with higher morbidity and mortality after discharge [6].

Some studies have found that tracheostomies improve the quality of life of long-term mechanically ventilated patients, reduce the need for analgesia and the rate of infections, and are associated with a reduction in mortality rates [9]. However, in contrast to adult patients [10], there is still reluctance to perform tracheostomies on long-term mechanically ventilated pediatric patients, with the timing for placement not yet established [9].

Pediatric tracheostomies are associated with a higher risk of complications and greater morbidity and mortality compared to adult tracheostomies [11]. Diagnostic endoscopic procedures, including laryngoscopies and bronchoscopies, are used to identify lesions that may subsequently result in complications or facilitate early tracheostomy decannulation. Nonetheless, there is a notable scarcity of scientific studies examining contemporary surveillance practices for patients with long-term tracheostomies, particularly within the pediatric population. The use of endoscopic screening varies among healthcare institutions [2].

According to a survey, while most clinicians perform annual endoscopic surveillance for children under 2 years of age with tracheostomies, a significant proportion only conduct such examinations prior to decannulation or do so for patients experiencing complications [12].

The German National Guideline for the Management of Chronic Respiratory Failure recommends annual surveillance assessments, primarily due to the risk of complications, such as ulceration or granuloma formation [13].

Unfortunately, only a small number of children with complex diseases have access to specialized pediatric centers that can carry out annual endoscopic surveillance. In 2022, we established an advanced care program to monitor these children closely. At our pediatric palliative care facility, we established two operating rooms to expand the diagnostic and therapeutic options available for children and young adults with complex medical conditions. We also ensured the availability of anesthesiologists with expertise in managing complex medical conditions to provide anesthesia support during these interventions.

In our specialized pediatric palliative care unit, patients are prepared for interventions and bronchoscopies with close post-procedural monitoring, allowing us to closely observe the outcomes of our interventions and make adjustments to our previous approaches as needed.

In this case report, we present four pediatric patients who developed tracheal complications after prolonged invasive mechanical ventilation. This study aims to highlight the importance of the early recognition and management of these complications to prevent adverse outcomes.

## 2. Case Presentations

The first case involved an 11-year-old female patient with severe hypoxic encephalopathy after birth and prolonged asphyxia. She underwent tracheostomy at one year of age and has been dependent on mechanical ventilation ever since. She had undergone routine assessments at a specialized medical institution until the age of 7 years. Due to her complex chronic illness, which required ventilation; neurological impairment; and gastrointestinal disorders, no such examinations were performed in the following 4 years.

The patient was transferred from the pediatric intensive care unit to our pediatric palliative care facility, where she was initially admitted for an assessment of her ventilatory requirements. The intensive care unit referred the patient to our facility after learning about our specialized surveillance program for children with complex medical conditions requiring long-term mechanical ventilation.

**Figure 1:**
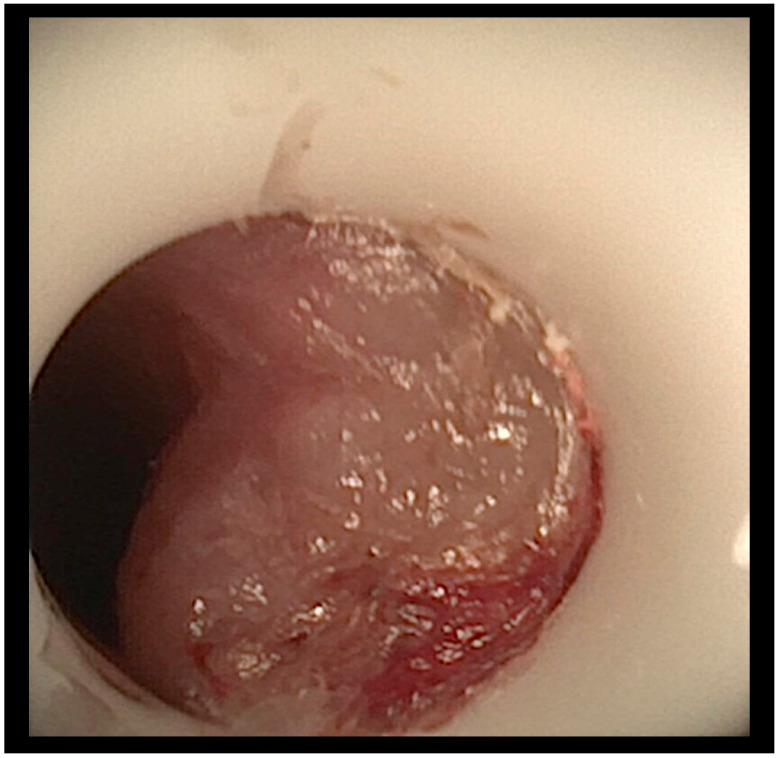
Tracheoscopic visualization of an obstructing granuloma at the distal end of the tracheostomy cannula.

A flexible bronchoscopic examination under general anesthesia demonstrated extensive ulcerative granulation tissue distal to the tracheostomy cannula obstructing 60% of the tracheal lumen (Figure 1). To address this finding, the tracheal cannula was replaced with a longer cannula extending beyond the granuloma. The insertion of the extended cannula was performed under bronchoscopic guidance. A subsequent follow-up evaluation at six months revealed a substantial reduction in the granuloma’s size.

The second case involved a 6-year-old boy who was diagnosed with a severe form of a nemaline myopathy and actin accumulation myopathy caused by a mutation in the ACTA1 gene. Due to respiratory failure, he has required long-term mechanical ventilation through a tracheostomy since the age of 18 months.

At 5 years of age, the patient experienced episodes of tracheal obstruction during ventilation and produced secretions mixed with blood upon suctioning. A rigid bronchoscopy performed by an otolaryngologist revealed the presence of a granuloma. A longer tracheostomy tube was ordered, and the patient received local triamcinolone therapy. However, his symptoms worsened; the patient experienced recurrent episodes of severe tracheal obstruction and desaturation, prompting referral to our pediatric palliative care facility.

**Figure 2:**
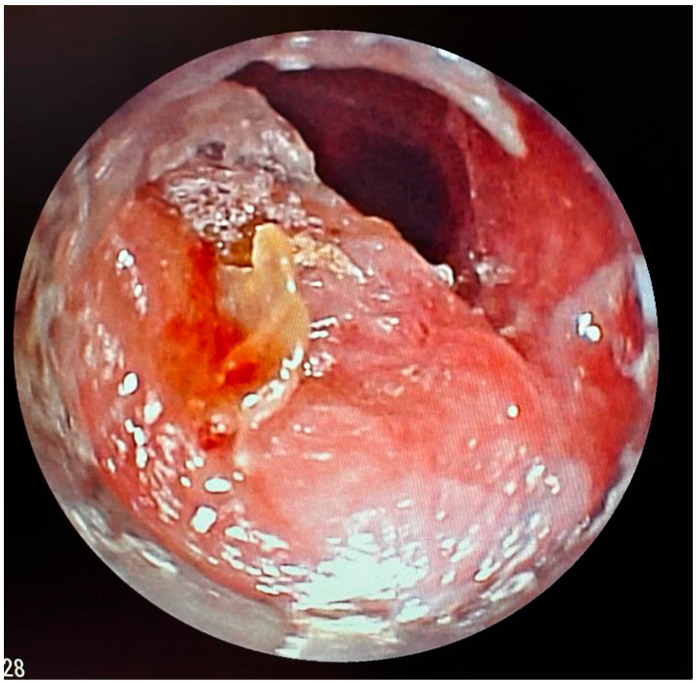
Tracheoscopic visualization at the distal end of the tracheostomy cannula. The cannula abrades the granuloma.

A flexible bronchoscopic examination at our facility showed extensive granulomatous tissue distal to the tracheostomy tube, with significant inflammation in the distal trachea and bronchi (Figure 2).

To address the recurrent tracheal obstruction and granulomatous tissue, we implemented a management approach. This involved shortening the tracheostomy tube and changing the size of the tube every two weeks to prevent further granulation tissue formation. Additionally, we conducted flexible bronchoscopic examinations every 8 weeks over a 6-month period to monitor the healing process and ensure proper airway patency.

**Figure 3:**
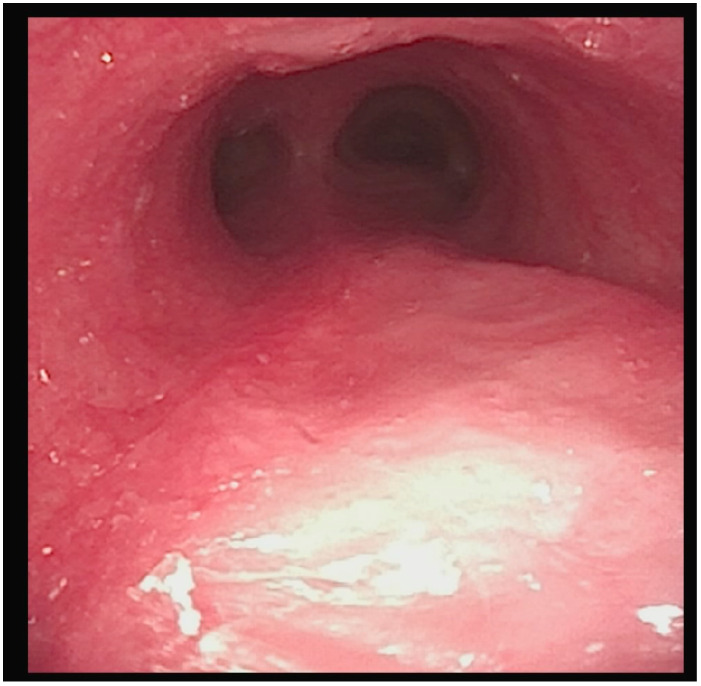
Image taken after 3 months of specific cannula management.

After this comprehensive approach, the patient’s tracheal obstruction episodes resolved, and the granulomatous tissue showed significant regression (Figure 3).

The third case involved a 9-year-old boy diagnosed with combined D2-/L2-hydroxyglutaric aciduria (SLC25A1 deficiency). He underwent a tracheostomy at 5 months of age and has required intermittent mechanical ventilation for the past 3 years. The patient underwent his first bronchoscopic examination at our center when he was 8 years old.

Our specialized pediatric palliative care team has been managing the patient’s care at home. They reported intermittent episodes of desaturation and ventilation-related issues; changing the tracheostomy tube had little effect. Due to the ineffectiveness of the interventions in the home setting, we planned to have the patient admitted to our facility and subsequently undergo a bronchoscopic evaluation.

**Figure 4:**
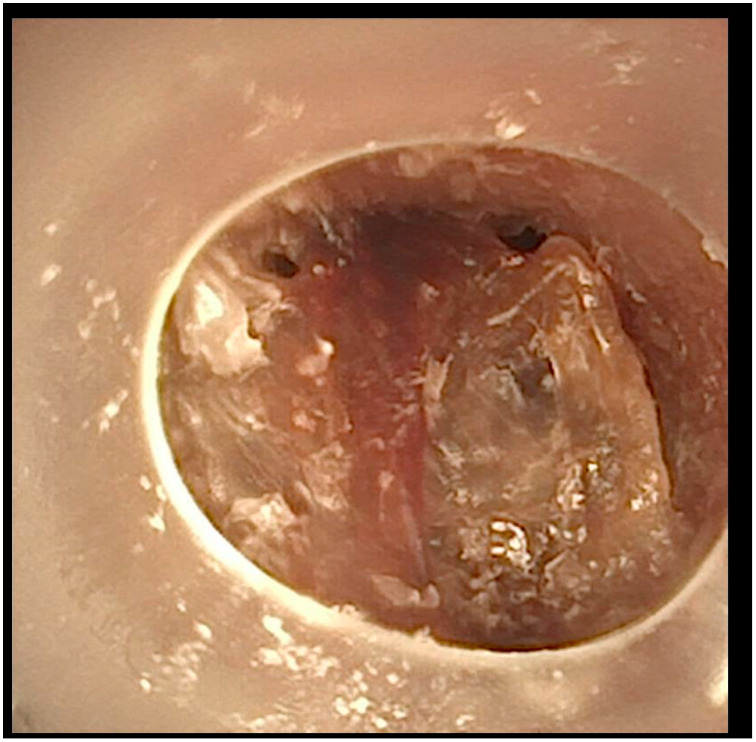
Mucus plug obstructing trachea.

**Figure 5:**
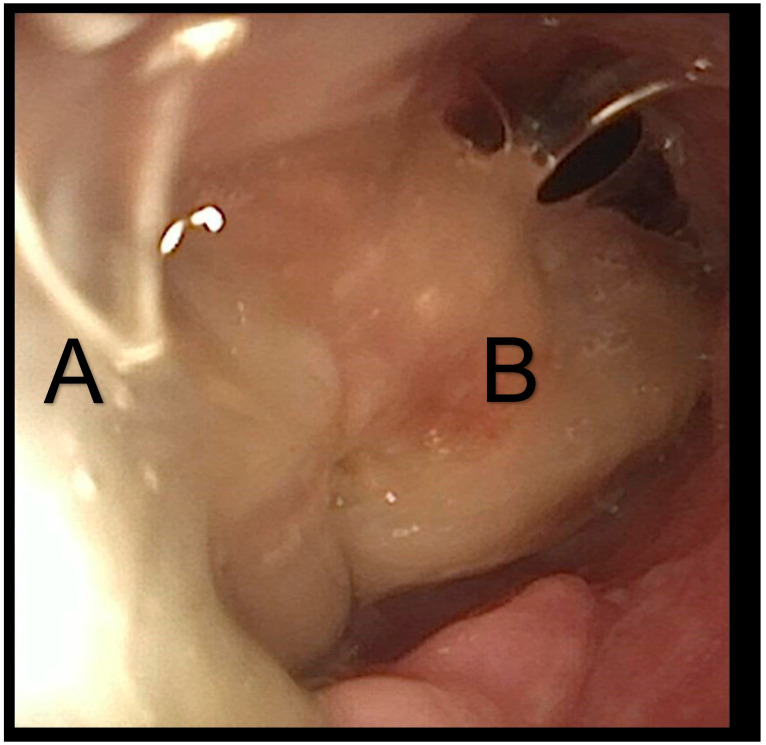
Withdrawal of the mucus plug. The opening made by the tracheostomy is visible on the left side (**A**). The cannula has been pulled back. A portion of the mucus plug remains within the trachea (**B**).

Bronchoscopic examination revealed a large mucus plug that obstructed the entire tracheal lumen and was distal to the tracheostomy tube (Figure 4). The mucus crust was larger than the tracheostomy tube, making retrieval very challenging. During extraction attempts, the plug shifted position and was found in both main bronchi. The patient experienced desaturations, necessitating the insertion of a cuffed tracheostomy tube and increased ventilatory support. Under bronchoscopic guidance, a large intratracheal suctioning device was utilized to successfully remove the large mucus plug (Figure 5). After thorough tracheal cleaning, the patient’s oxygen saturation improved to 95%. A subsequent bronchoscopy showed no significant pathology or residual mucus.

As a follow-up measure, the patient underwent Dornase alfa inhalation therapy for three days, after which no further issues were reported.

The fourth case involved a 19-year-old female patient diagnosed with lissencephaly, a severe form of cerebral palsy, and epilepsy. Due to her substantial neurological impairment, the patient required a tracheostomy and mechanical ventilation from her first year of life. The patient, although 19 years old, has been treated within a pediatric setting due to her need for long-term ventilation since early childhood and body size/weight of 30 kg, aligning with pediatric criteria. During her first 12 years, she underwent regular follow-up examinations conducted by a local pulmonologist.

Following relocation to another area in Germany, the patient no longer underwent regular follow-up examinations. Her family independently managed her ventilation parameters, inserted a blocked cannula, and escalated ventilation settings.

At 19 years of age, the patient experienced a sudden clinical decline, exhibiting ventilation, desaturation, vomiting, and an inability to tolerate oral intake of food. She was then transferred to an adult healthcare facility, where gastroscopic examination revealed pangastritis, whereas bronchoscopic evaluation did not reveal any pathological findings.

The symptoms exacerbated over the subsequent four weeks, and the patient was referred to our pediatric palliative care facility.

The individual presented in a critical condition, exhibiting severe dehydration and profound hypokalemia (2.0 mmol/L). Despite intensifying ventilatory support with high inspiratory pressure, adequate oxygenation was not achieved. Radiographic imaging of the thorax revealed signs of subcutaneous emphysema in the neck region and the presence of air within the mediastinum.

**Figure 6:**
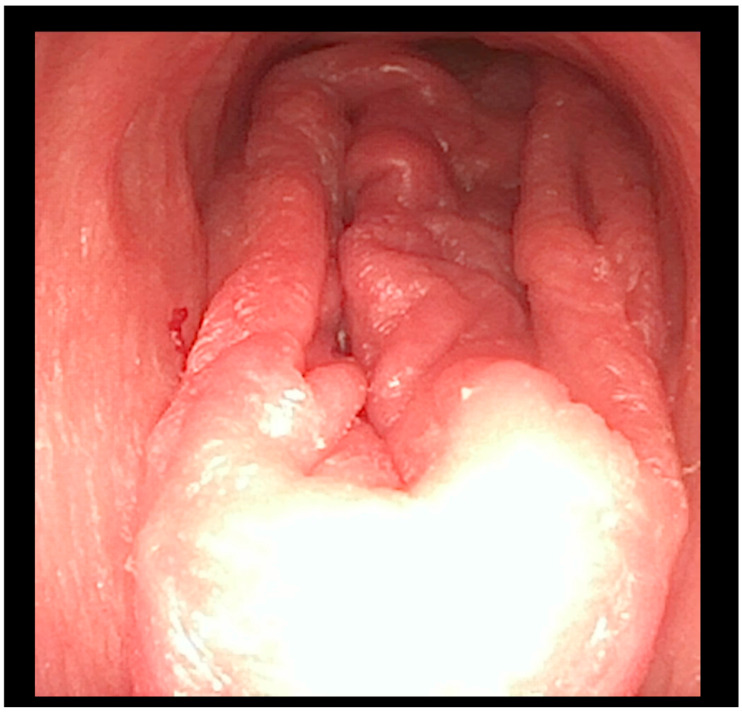
View of the deformed posterior tracheal wall, with large, deep protrusions.

**Figure 7:**
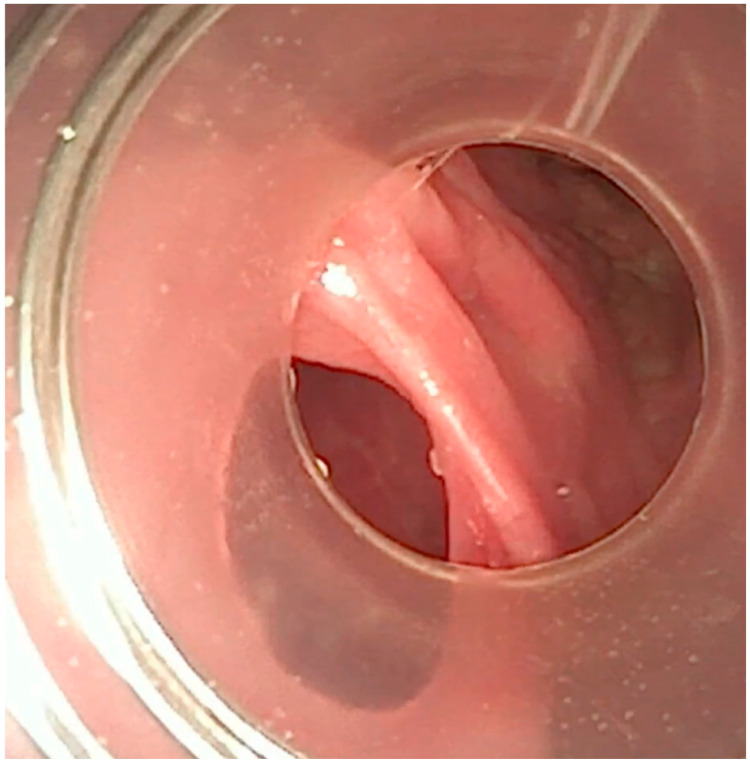
View through the distal end of the tracheostomy cannula. In the upper right quadrant, the trachea, with its posterior wall, is visible, while in the lower left quadrant, the fistula leading to the esophagus can be observed.

We established a central line and conducted a flexible bronchoscopy under general anesthesia. The bronchoscopy revealed significant deformation of the posterior tracheal wall, with the formation of large, deep protrusions (Figure 6). Upon retracting the tracheostomy tube, a 5 mm defect was identified in the posterior wall of the trachea. The bronchoscope could pass through this defect, leading to the esophagus, and a tracheoesophageal fistula was found (Figure 7).

Due to the patient’s underlying diagnosis and life expectancy, extensive discussions were held with her parents. Collaboratively, we established the goal of facilitating the child’s return home and providing subsequent care in that environment. We determined that transferring the patient to the pediatric surgery department would be the appropriate course of action, where the tracheoesophageal fistula was subsequently closed through surgical intervention.

Following surgical intervention, the patient was transferred to the pediatric palliative care unit. The patient’s respiratory status improved, and oral feeding was resumed. Subsequently, the patient was discharged to her home environment with enhanced quality of life.

Despite adjusting the tracheostomy tube to a non-cuffed type and reducing ventilatory pressure, the patient subsequently experienced a recurrence of the tracheal fistula within a few months. Given the patient’s complex chronic condition and the chronically deformed trachea resulting from prolonged mechanical ventilation with a cuffed tube and high pressure, a palliative approach at home was determined to be the appropriate course of action. The patient died in her home environment with the support of a pediatric palliative care team.

## 3. Discussion

These four case reports illustrate the diverse range of tracheal complications that can arise in pediatric patients requiring long-term mechanical ventilation, including granuloma formation, tracheomalacia, obstruction, and tracheoesophageal fistulas [13].

According to the literature reviewed, the complications observed in these case reports are relatively rare. Gergin et al. [3] conducted a surveillance study of 303 patients with tracheostomies and found that 55.1% were diagnosed with a single lesion or a combination of lesions, with the most common being minor issues like suprastomal granulation, suprastomal collapse, and peristomal granulation. More severe lesions, such as distal tracheal granulation and tracheal ulceration, were observed in 4.9% and 1.3% of patients, respectively. Similarly, Sharif-Askary et al. [1] reported tracheal ulcerations in 3.3% of their study population.

The first two cases presented in this paper demonstrated tracheal ulcerations with distal granulomas, rare complications, which may be attributed to the extended time interval between the tracheostomy and the first bronchoscopic evaluation. However, our literature review indicates that the time differential between the tracheostomy and initial surveillance bronchoscopy has decreased over the years. Sharif-Askary et al. [1] found that the median time between a tracheostomy and first bronchoscopy was 13 months, with a median interval of 7 months between subsequent bronchoscopies. Additionally, the proportion of patients with airway findings who underwent bronchoscopy within 6 months post-tracheostomy was significantly lower compared to those who were subjected to it after 6 months.

While annual surveillance laryngobronchoscopy carries risks such as anesthetic complications and financial burdens, it remains an important tool in managing pediatric patients with chronic tracheostomies. The variability in current care practices across institutions and regions may impact reimbursement and the quality of patient care.

The American Thoracic Society published consensus clinical practice guidelines 25 years ago, recommending a routine rigid or flexible bronchoscopy every 6 to 12 months for pediatric tracheostomy management [14]. To the best of our knowledge, no new practical guidelines have been established since then. The cited authors emphasized the importance of these assessments for evaluating underlying airway pathology, managing complications, ensuring proper tube fit and placement, and assessing decannulation readiness. They also noted that more frequent endoscopic evaluations may be necessary for patients with unstable health or accelerated growth. Recognizing that many recommendations were based on consensus in the absence of definitive scientific evidence, the authors highlighted the importance of further research into the utility and optimal timing of surveillance endoscopies. Consequently, the German National Guideline for the Management of Chronic Respiratory Failure advises yearly surveillance bronchoscopic evaluations, mainly because of the potential for complications like ulceration or granuloma development [13]. As a result, the standard of care at our institution is to conduct annual surveillance bronchoscopies on all asymptomatic patients.

This case series presents limited and informal data, so it cannot be used to justify the use of routine endoscopic examinations for patients without symptoms. Moreover, it does not suggest that an annual endoscopy would decrease the incidence or severity of tracheostomy complications. The ability of an endoscopy to prevent complications before clinical manifestations is unclear; therefore, a general recommendation cannot be made based on this case series, despite the logical appeal.

The third case demonstrated a severe, life-threatening tracheal obstruction caused by a mucus plug. Mild obstructions are prevalent in the care of children with tracheostomy. Tracheostomy tubes circumvent the natural processes of air filtration and humidification that typically occur in a child’s upper respiratory tract. Consequently, children with tracheostomies may experience increased secretion viscosity, necessitating the use of humidification and more frequent suctioning to mitigate the risk of tracheal tube obstructions [15].

Despite the caregivers’ efforts to mobilize secretions and employ mechanical insufflator–exsufflator therapy in the preceding weeks, a substantial obstruction persisted. The severity of this condition was not recognized as potentially life-threatening.

According to a survey by McCormick et al. [4], the most common serious problems associated with tracheostomy care reported by families are mucus plugging (18.2%) and accidental decannulation (17.6%). Mucus plugging can cause serious complications. In one Canadian study [15], mucus plugs formed in-hospital led to cardiopulmonary arrest in seven patients (10%) within 7–90 days post-tracheotomy. This complication predominantly affected young patients. All affected individuals experienced cardiorespiratory arrest, with one fatality.

Children with tracheostomies necessitate comprehensive care from parents and medical staff within their home environment. Routine procedures encompass tracheostomy tape changes, suctioning, manual ventilation, infection control measures, stoma care, and emergency preparedness. One study [16] revealed multiple issues concerning the care processes for children undergoing long-term ventilation in community settings. A problem was identified in each incident, with some incidents involving two or three issues. Ninety-one incidents related to procedural issues, including dislodged tracheostomy tubes, incorrect adherence to protocols, the use of inappropriate-sized tracheostomy tubes, and problems associated with tracheostomy tapes. Twenty-seven incidents raised concerns about staff, including parental concerns regarding staff competence, staff falling asleep while caring for children, staff unavailability, and staff-training inadequacies. Communication breakdowns between staff members or between staff and families were reported in 18 incidents. The analysis indicated clear harm to children in 89 incidents, while the remaining 131 incidents represented potential harm or cases where harm was not explicitly stated. The outcomes for children varied in severity, with some incidents requiring immediate emergency intervention.

The final case presented a severe, life-threatening tracheoesophageal fistula. Tracheoesophageal fistulas following tracheostomy placement are more often referred to as a tracheostomy complication in adults and are uncommon in pediatric populations. These fistulas typically occur due to necrosis of the posterior tracheal wall, often secondary to the use of poorly positioned tracheostomy tubes with cuffs. In one study evaluating the effectiveness of a sliding tracheoplasty for repairing tracheoesophageal fistulas, one of the nine operated cases was attributed to complications arising from tracheostomy tube erosion [17].

Two of the cases underwent bronchoscopy at an external medical facility shortly before presenting to our institution. The first child did not receive appropriate management, whereas the latter was misdiagnosed as exhibiting a severe complication of a tracheoesophageal fistula. This evidence demonstrates that children with complex diseases require treatment at a specialized pediatric facility using a comprehensive approach.

Moreover, all the cases emphasize the importance of comprehensive follow-ups and education for families managing complex tracheostomies at home. These case reports underscore the need for close monitoring and a multidisciplinary approach in the care of pediatric patients with long-term tracheostomies to prevent potentially life-threatening complications.

Healthcare professionals working with ventilator-dependent children with tracheostomies should be aware that these patients require regular annual bronchoscopies. Implementing this approach could be challenging because of limited resources and the fact that children with complex diseases and long-term ventilation are not easily transportable. German guidelines for Chronic Respiratory Failure [13] emphasize complications linked to cuffed cannulas in pediatric patients. In light of limited resources, extending the surveillance period for asymptomatic children using uncuffed cannulas could be a viable strategy. The limited use of cuffed cannulas presents a chance to enhance tracheostomy maintenance and potentially minimize the requirement for bronchoscopies. One approach might involve an annual bronchoscopy for children with cuffed tubes, given the difficulties in home care. A low threshold for diagnostic bronchoscopy should be considered when recurring issues arise.

Consequently, there is a need for additional centers that provide facilities where children with complex diseases can undergo regular surveillance as well as interventions during complications. These centers should also possess the capability to implement a comprehensive approach in palliative care due to the complexity of the conditions. This approach addresses the physical, emotional, social, and spiritual needs of both the children and their families. This comprehensive care model emphasizes enhancing quality of life and providing comfort rather than solely treating the underlying condition—in this case, the problem with the trachea. It involves a multidisciplinary team of healthcare professionals, including physicians, nurses, social workers, psychologists, and spiritual counselors, who collaborate to manage symptoms, alleviate complications, and provide emotional support. Palliative care for children extends beyond medical interventions, encompassing age-appropriate activities and the education of both parents and healthcare providers. This holistic approach ensures that all aspects of a child’s and family’s well-being are considered and supported throughout the course of their illness and beyond.

We would like to recommend that pediatric palliative care centers deliver comprehensive care to these patients and their families. Currently, not all pediatric palliative care centers possess the resources necessary to provide medical assistance for long-term ventilation and tracheostomy management. This care should include access to specialized facilities and staff with the expertise to perform and interpret bronchoscopy examinations within a holistic approach to better address the needs of children and their families. We must stress that the exclusive provision of care at highly specialized centers lacks supporting evidence. If such specialized care is unavailable, it is crucial that patients and their families have access to facilities with experience in long-term ventilation, tracheostomy management, and bronchoscopy.

## Data Availability

The original contributions presented in the study are included in the article; further inquiries can be directed to the corresponding author.
